# The structure of the cysteine protease and lectin-like domains of Cwp84, a surface layer-associated protein from *Clostridium difficile*

**DOI:** 10.1107/S1399004714009997

**Published:** 2014-06-29

**Authors:** William J. Bradshaw, Jonathan M. Kirby, Nethaji Thiyagarajan, Christopher J. Chambers, Abigail H. Davies, April K. Roberts, Clifford C. Shone, K. Ravi Acharya

**Affiliations:** a Department of Biology and Biochemistry, University of Bath, Claverton Down, Bath BA2 7AY, England; b Public Health England, Porton Down, Salisbury SP4 0JG, England

**Keywords:** *Clostridium difficile*, surface layer-associated protein, Cwp84

## Abstract

*Clostridium difficile* is a major problem as an aetiological agent for antibiotic-associated diarrhoea. The mechanism by which the bacterium colonizes the gut during infection is poorly understood, but undoubtedly involves a myriad of components present on the bacterial surface. The mechanism of *C. difficile* surface-layer (S-layer) biogenesis is also largely unknown but involves the post-translational cleavage of a single polypeptide (surface-layer protein A; SlpA) into low- and high-molecular-weight subunits by Cwp84, a surface-located cysteine protease. Here, the first crystal structure of the surface protein Cwp84 is described at 1.4 Å resolution and the key structural components are identified. The truncated Cwp84 active-site mutant (amino-acid residues 33–497; C116A) exhibits three regions: a cleavable propeptide and a cysteine protease domain which exhibits a cathepsin L-like fold followed by a newly identified putative carbohydrate-binding domain with a bound calcium ion, which is referred to here as a lectin-like domain. This study thus provides the first structural insights into Cwp84 and a strong base to elucidate its role in the *C. difficile* S-layer maturation mechanism.

## Abstract 2

The crystal structure of Cwp84, an S-layer protein from *Clostridium difficile* is presented for the first time. The cathepsin L-like fold of cysteine protease domain, a newly observed ‘lectin-like’ domain and several other features are described.

## 1. Introduction   

Disruption of the normally protective gut flora results in the extensive colonization and growth of *Clostridium difficile* (Guarner & Malagelada, 2003 ▶), a predominantly nosocomially acquired Gram-positive, spore-forming bacterium. *C. difficile* infection (CDI) can lead to severe diarrhoea, pseudo­membranous colitis, toxic megacolon and ultimately death (Kachrimanidou & Malisiovas, 2011 ▶; Rupnik *et al.*, 2009 ▶). In recent years, CDI has become a global burden both medically and economically (Bouza, 2012 ▶; Dubberke & Olsen, 2012 ▶).

*C. difficile* expresses a self-assembling paracrystalline protein array on its outermost surface, known as an S-layer. The S-layer is largely derived from the post-translational cleavage of a single polypeptide (surface-layer protein A; SlpA) into low- and high-molecular-weight subunits (LMW SLP and HMW SLP, respectively) by Cwp84, a surface-located cysteine protease (Calabi *et al.*, 2001 ▶; Cerquetti *et al.*, 2000 ▶; Karjalainen *et al.*, 2001 ▶; Kirby *et al.*, 2009 ▶).

The HMW SLP contains three putative cell-wall binding/anchoring domains (CWBDs; Pfam 04122) which are thought to mediate noncovalent binding to the bacterial cell surface *via* a currently unknown mechanism. A total of 28 S-layer paralogues, including Cwp84, containing three Pfam 04122 repeats at either the N-terminal or C-terminus with a ‘functional’ domain at the other end, have been identified in the *C. difficile* genome (Calabi *et al.*, 2001 ▶; Fagan *et al.*, 2011 ▶; Monot *et al.*, 2011 ▶; Sebaihia *et al.*, 2006 ▶).

A number of these putative surface proteins have been found to play key roles in cell physiology and adhesion (Kirby *et al.*, 2009 ▶; Reynolds *et al.*, 2011 ▶; Waligora *et al.*, 2001 ▶), and have been demonstrated to illicit an immune response *in vivo* during infection (Wright *et al.*, 2008 ▶). Using the ClosTron gene-knockout system, we have demonstrated that a number of *C. difficile* surface-associated genes containing Pfam 04122 repeats may play a role in adhesion *in vitro* and may also affect the release of the potent *C. difficile* toxins, particularly Cwp84 (Kirby *et al.*, unpublished work).

**Figure 1:**
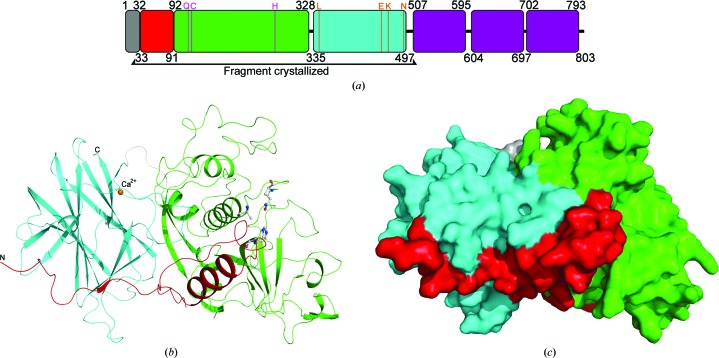
(*a*) Domain structure of full-length Cwp84. The domains are indicated as follows: signal peptide, grey; propeptide, red; cysteine protease, green; lectin-like, cyan; CWBDs, purple. Active-site residues are indicated in pink, while calcium ion-coordinating residues are shown in orange. The region crystallized, consisting of residues 33–497, is bracketed below. (*b*) Ribbon diagram of the three-dimensional structure of the propeptide, cysteine protease and lectin-like domains. The domains are coloured according to (*a*) and the calcium ion is represented as an orange sphere. The disordered region between Lys81 and Tyr89 can be observed as a discontinuity in the ribbon at the bottom centre of the image. (*c*) Molecular surface of Cwp84_33–497_. The close interaction of the propeptide with the cysteine protease and lectin-like domains is shown, particularly at the active site formed at the interface between the cysteine protease and lectin-like domains. The domains are coloured according to (*a*).

Cwp84 (cell-wall protein ∼84 kDa) is an 803-residue surface-associated protein containing a cysteine protease domain at the N-terminus, a linker region of roughly 170 residues of unknown function and three Pfam 04122 repeats (Fig. 1 ▶ *a*; Janoir *et al.*, 2004 ▶, 2007 ▶). Cwp84 has been shown to be responsible for the maturation of the SlpA precursor protein (Dang *et al.*, 2010 ▶; de la Riva *et al.*, 2011 ▶; Kirby *et al.*, 2009 ▶) and has also been implicated in the degradation of extracellular matrix proteins such as fibronectin, laminin and vitronectin (Janoir *et al.*, 2007 ▶).

Despite the key role played by Cwp84 in S-layer biogenesis, it has been reported that neither chemical inhibition of Cwp84 (Dang *et al.*, 2010 ▶) nor inactivation of the *cwp84* gene (de la Riva *et al.*, 2011 ▶; Kirby *et al.*, 2009 ▶) is bactericidal, although severe growth defects were seen in both cases. These results indicate that correct maturation of SlpA by Cwp84 is vital to maintain healthy bacterial cells; perturbing this process may therefore affect the ability of the bacterium to thrive *in vivo* and thus compete with other bacterial species in certain environments, such as in the complex microbiome of the intestine. Nevertheless, in a hamster model of acute infection we previously showed that a *cwp84* knockout strain of *C. difficile* was not attenuated for virulence and suggested that endogenous proteases within the intestinal tract may artificially mature/cleave SlpA (Kirby *et al.*, 2009 ▶). However, our unpublished observations suggest that *C. difficile* toxin release is altered in the *cwp84* mutant, which may negate severe growth defects (Kirby *et al.*, unpublished work). Even so, it has been speculated that the interruption of S-layer biogenesis may make the bacterium more susceptible to antibiotics (Dang *et al.*, 2010 ▶). This makes Cwp84 a potential target for novel prophylactic or therapeutic drugs against CDI, the development of which would be guided by structural analyses of the protein.

Cwp84 is a member of the C1A cysteine protease family (Rawlings *et al.*, 2010 ▶), also known as papain proteases, with a putative catalytic dyad comprising of residues Cys116 and His262, aided by Asn287 (Savariau-Lacomme *et al.*, 2003 ▶). Recently, Dang and coworkers showed that Cwp84 containing the substitution Cys116Ala did not cleave SlpA in an *Escherichia coli*-based co-expression assay, confirming that Cys116 is a catalytically important residue (Dang *et al.*, 2010 ▶). Papain peptidases are typically composed of an N-terminal signal peptide, a propeptide and the catalytic domain. After the removal of the signal peptide by a signal peptidase, the proenzyme often (but not always; Dahl *et al.*, 2001 ▶; Nägler *et al.*, 1999 ▶) undergoes self-cleavage, removing the proregion and generating the mature, active enzyme (Beton *et al.*, 2012 ▶; ChapetónMontes *et al.*, 2011 ▶). It has been proposed that the propeptide ensures the correct folding of the protein (ChapetónMontes *et al.*, 2011 ▶). A recent study by de la Riva and coworkers showed that Cwp84 is produced as an inactive proenzyme and is processed into the active enzyme of 77 kDa by removal of the signal peptide and proregion up to Ser92 and that this activation step is unlikely to be autocatalytic (de la Riva *et al.*, 2011 ▶).

Despite adherence and subsequent colonization by *C. difficile* representing key milestones in infection, there are considerable gaps, particularly with regard to structural data, in the understanding of how the surface proteins of *C. difficile* interact with each other and their environment. To date, there has only been one previous report of structural information for a *C. difficile* surface protein, which presented the crystal structure of an N-terminal fragment of the low-molecular-weight subunit of the S-layer at 2.4 Å resolution (PDB entry 3cvz) and structures based on solution-scattering (SAXS) experiments of both full-length LMW SLP and the complex formed by LMW SLP and HMW SLP (Fagan *et al.*, 2009 ▶).

To further the understanding of *C. difficile* S-layer biogenesis, we report a high-resolution (1.4 Å) crystal structure of the N-terminal cysteine protease domain of Cwp84. Interestingly, the hitherto uncharacterized 170-residue ‘linker’ region between the cysteine protease domain and putative location of the first Pfam 04122 repeat exhibits a lectin-like domain structure with a bound calcium ion.

## 2. Materials and methods   

### 2.1. Protein expression and purification   

A synthetically synthesized gene encoding *C. difficile* Cwp84 residues 33–497 (from strain QCD32g-58; ribotype 027) with a C116A mutation (an inactive mutant; Life Technologies GeneArt Ltd) was cloned by PCR into the GST expression vector pGEX-6P-1. The mutation was introduced to potentially circumvent problems with poor expression and degradation or problems with purification (based on initial trials with multiple constructs designed without the mutation). Of the two constructs produced with the mutation, neither had the problems discussed above and one was purified to near-homogeneity in one step (see below). The structure presented in this manuscript made use of this particular construct.

The gene was amplified from the stock pMA vector by PCR with Expand High Fidelity polymerase (Roche) utilizing primers incorporating cleavage sites for *Bam*HI at the 5′ end and *Not*I at the 3′ end preceded by a TAA stop codon (forward primer GAGAGTCCTCGGATCCCACAAAACC­CTGGATGGCGTGGAA, reverse primer CTCTCTCGCG­GCCGCTCTTAGCTGGTTTTGGTGATCGCTT). The PCR products were digested with *Bam*HI and *Not*I (NEB) and cloned into pGEX-6P-1 using T4 DNA ligase (New England Biolabs) to generate pGEX-6P-1-Cwp84_33–497_C116A.

The plasmid was transformed into *E. coli* BL21*(DE3) cells. Cultures were grown from glycerol stocks in 5 ml LB supplemented with 100 µg ml^−1^ ampicillin for 17 h and centrifuged (5000*g*, 10 min). The cell pellets were washed with water, centrifuged a second time, resuspended in water and used to inoculate 500 ml selenomethionine medium (Molecular Dimensions) supplemented with 100 µg ml^−1^ ampicillin. These cultures were grown with shaking (200 rev min^−1^, 37°C) to an OD_600_ of 0.7. The temperature was reduced to 16°C and methionine production was inhibited by the addition of 100 µg ml^−1^ lysine, phenylalanine and threonine and 50 µg ml^−1^ leucine, isoleucine and valine. 60 µg ml^−1^ selenomethionine was also added and the cultures were incubated for 15 min before expression was induced with 1 m*M* IPTG. The cultures were incubated for a further 18 h and harvested by centrifugation (8000*g*, 10 min).

The cell pellets were resuspended in PBS (140 m*M* NaCl, 2.7 m*M* KCl, 5 m*M* DTT, 10 m*M* Na_2_HPO_4_, 1.8 m*M* KH_2_PO_4_ pH 7.3), lysed in a French press and clarified by centrifugation (75 000*g*, 25 min). The supernatant was loaded onto a GSTrap column (GE Healthcare) and washed with PBS, and tagged protein was eluted with 10 m*M* glutathione, 50 m*M* Tris–HCl pH 8.0. PreScission protease (80 µl) was added and the eluted protein was dialyzed overnight into cleavage buffer (50 m*M* Tris–HCl, 150 m*M* NaCl, 1 m*M* EDTA, 5 m*M* DTT pH 7.5). The dialyzed sample was then reloaded onto the GSTrap column to separate the unbound protein from the tag.

Unbound protein was concentrated to a volume of roughly 1 ml and further purified by size-exclusion chromatography (SEC) into 50 m*M* Tris–HCl pH 8.0 (using a Superdex 200 16/600 column); fractions containing Cwp84_33–497_C116A were pooled and concentrated to 11.9 mg ml^−1^.

### 2.2. Trypsin cleavage of Cwp84   

GST-Cwp84_33–497_ was incubated with trypsin at a molar ratio of approximately 10:1 for 45 min. Following purification by SEC in 25 m*M* MOPS pH 7.0, the resulting single species (Cwp84_92–497_) was analysed by electrospray ionization mass spectrometry. Cwp84_92–497_ was also transferred onto PVDF and sent for N-terminal sequencing (AltaBioscience).

### 2.3. X-ray crystallographic studies   

Crystallization-condition screening was performed with a range of pre-prepared 96-well screens (Molecular Dimensions) using an Art Robbins Phoenix nanodispensing robot. Optimal conditions were reproduced with 0.3 µl drops with a 1:1 ratio of protein to reservoir solution (0.2 *M* ammonium sulfate, 30% PEG 4K; Molecular Dimensions Structure Screen 1 & 2, solution D7). Crystals took between 3 d and a week to grow.

X-ray diffraction data were collected at station I03 at Diamond Light Source (DLS; Didcot, Oxfordshire, England). The diffraction data were recorded with 1.0° oscillation on a Pilatus 6M detector from four crystals to obtain maximum redundancy. Selenium-fluorescence peak and inflection data were collected from all four crystals (to a maximum resolution of 1.73–1.87 Å), while high and low remote data were collected from two crystals (to a maximum resolution of 1.94–2.16 Å). 1120 peak images were collected at 12 660 eV, 1120 inflection images at 12 656 eV, 540 low-remote images at 12 550 eV and 540 high-remote images at 12770 eV. The data were automatically indexed and integrated with *XDS* (Kabsch, 2010 ▶) and *xia*2 (Winter *et al.*, 2013 ▶), respectively. The data were scaled (and resolutions cut to those reported in Table 1 ▶ to reduce errors) with *SCALA* (Diederichs & Karplus, 1997 ▶), combined with *CAD* (*CCP*4; Winn *et al.*, 2011 ▶) and put into the *Crank* MAD pipeline (*CCP*4; Ness *et al.*, 2004 ▶) with a resolution cutoff of 2.5 Å using *SCALEIT* (Howell & Smith, 1992 ▶), *AFRO* (*CCP*4), *CRUNCH*2 (de Graaff *et al.*, 2001 ▶), *BP*3 (Pannu *et al.*, 2003 ▶; Pannu & Read, 2004 ▶), *SOLOMON* (Abrahams & Leslie, 1996 ▶) and 500 cycles of *Buccaneer*/*REFMAC* (Cowtan, 2006 ▶; Murshudov *et al.*, 2011 ▶). *CRUNCH*2 found 55 potential selenium sites out of a predicted 48 within the unit cell, the validity of which was determined with the later programs, allowing *Buccaneer* and *REFMAC* to produce an output model with a figure of merit of 85.6% and *R*_cryst_ and *R*_free_ values of 24.8 and 27.7%, respectively. The model was further refined with *Coot*/*REFMAC* (Emsley & Cowtan, 2004 ▶) using a 1.4 Å resolution native data set collected on a Pilatus 6M on I02 at DLS that had been autoprocessed with *XDS* and *xia*2 and scaled with *AIMLESS* (Evans, 2006 ▶). Secondary structure was determined using *DSSP* (Kabsch & Sander, 1983 ▶) and the model was verified with *MolProbity* (Chen *et al.*, 2010 ▶).

The atomic coordinates and structure-factor amplitudes have been deposited with the RCSB Protein Data Bank (http://www.pdb.org) under PDB accession code 4ci7.

## 3. Results   

### 3.1. Overview   

**Figure 2:**
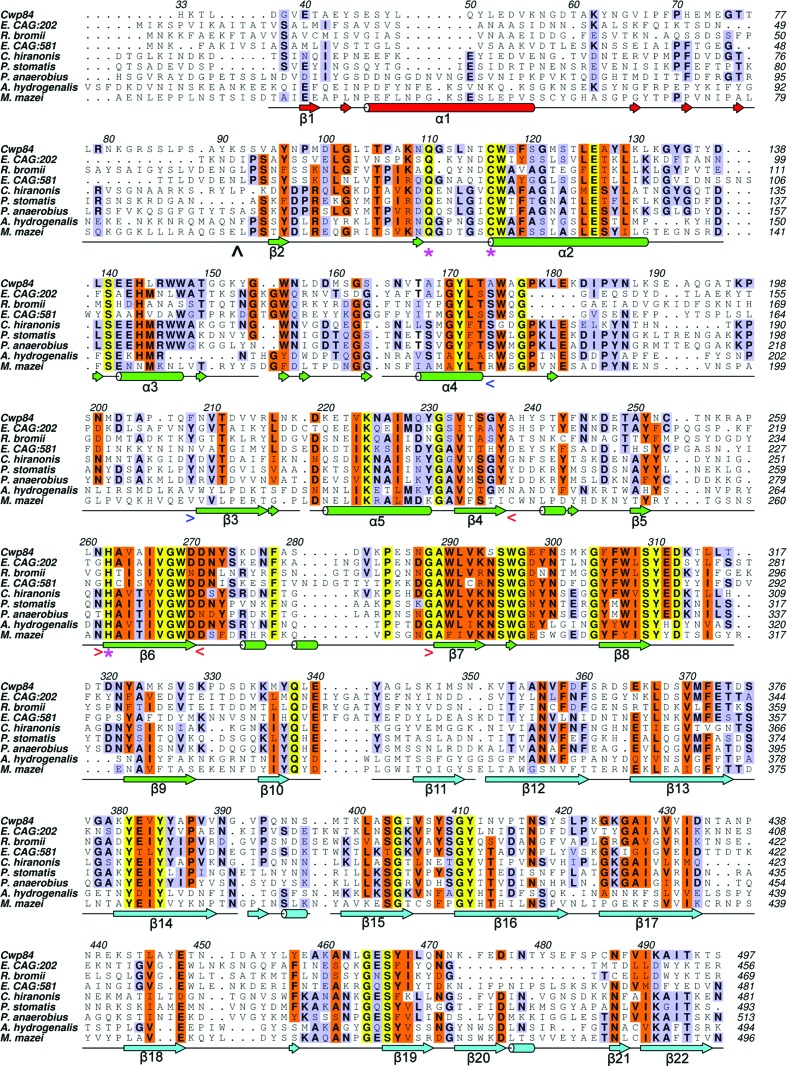
Multiple sequence alignment of Cwp84_33–497_ and the highest unique *BLAST* results. All are cysteine proteases that possess a putative lectin-like domain. The alignment was performed using *ClustalW*2 (Larkin *et al.*, 2007 ▶) and rendered with *ALINE* (Bond & Schüttelkopf, 2009 ▶). Strictly conserved residues are shown in yellow, medium to well conserved residues are in orange and slightly conserved residues are in blue. The secondary structure of Cwp84, as predicted by *DSSP* (Kabsch & Sander, 1983 ▶), is also shown coloured according to Fig. 1 ▶. 3_10_-Helices and β-bridges are displayed in the same way as α-;helices and β-strands, but are not numbered. Active-site residues (Gln110, Cys116 and His262) are indicated with pink stars, the propeptide cleavage site (Lys91-Ser92) is indicated with a black arrow and the occluding loop and PBL regions are indicated with blue and red triangular brackets, respectively. Sequences are taken from the following NCBI GenBank references: Cwp84, NC_009089; *Eubacterium* CAG:202, CDC03302; *Ruminococcus bromii*, YP_007780613; *Eubacterium* CAG:581, CDF12829; *Clostridium hiranonis*, WP_006441026; *Peptostreptococcus stomatis*, WP_007788460; *P. anaerobius*, WP_002842957; *Anaerococcus hydrogenalis*, WP_004816163; *Methanosarcina mazei*, NP_632235. The proteins from *C. hiranonis*, *P. stomatis* and *P. anaerobius* possess three putative Pfam 04122 repeats and thus are likely to be S-layer proteins performing similar functions to Cwp84.

We have determined the crystal structure of a truncated Cwp84 active-site mutant, residues 33–497, which comprises the propeptide, the cysteine protease domain and the newly identified ‘lectin-like’ domain (Fig. 1 ▶). This combination of a cysteine protease domain and a ‘lectin-like’ domain appears to be present in a number of species within the Clostridiales order and is also seen in a small number of archaea (Fig. 2 ▶), as revealed by a *BLASTP* search using Cwp84_33–497_ from strain 630, suggesting conservation of this particular domain arrangement. *DALI* searches using the whole structure did not reveal any proteins within the PDB with structural similarity over both domains.

**Figure 3:**
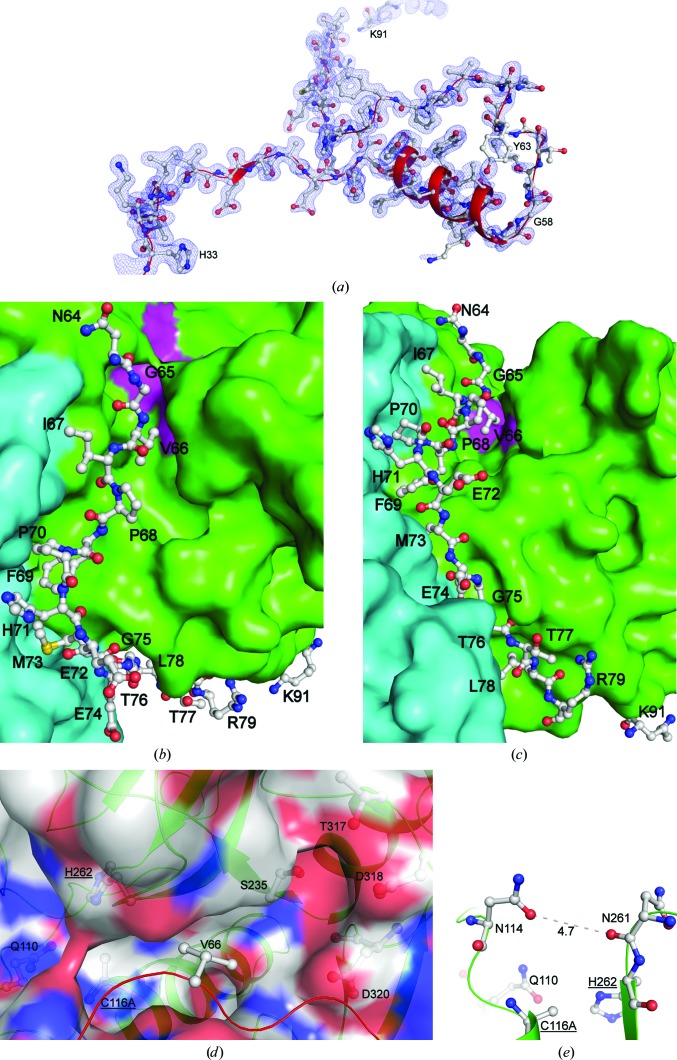
Cysteine protease propeptide and active-site groove. (*a*) The full length of the propeptide from His33 to Lys91 shown with sticks, ribbon and electron density (1σ, 2*F*_o_ − *F*_c_ map). The novel fold of the 30 residues is shown at the bottom of the image, while the normal section within the active-site groove is shown at the top of the image. Poor density that allowed modelling of Gly58–Tyr63 with a fair level of confidence can be observed on the right, and a lack of density for the unmodelled section towards the end of the propeptide is shown at the top. (*b*, *c*) Molecular surface of the cysteine protease active-site groove containing the propeptide; the two images are 50° apart. As in Fig. 1 ▶(*a*), the cysteine protease domain is shown in green and the lectin-like domain is shown in cyan; the three active-site residues are shown in pink. Propeptide residues before Asn64 have been removed for clarity. Met73 shows multiple conformations. Owing to the proximity of the side-chain carbonyl of Asn114 and the backbone carbonyl of Asn261 (4.7 Å in chain *A* and 4.6 Å in chain *B*), a continuous section of surface is shown above the active site. The propeptide fills the active-site groove and is shown in close contact with both domains. (*d*) Active site of Cwp84, with catalytic residues, residues involved in the formation of the S_2_ negatively charged pocket and Val66 from the propeptide shown. The negatively charged S_2_ pocket is shown surrounded by the residues that form it: Ser235, which shows multiple conformations, Thr317, Asp318 and Asp320. Note that Val66 does not enter the negatively charged pocket, but we propose that the P_2_ lysine of SlpA would. The oxyanion hole, formed by Gln110 and Cys116Ala, which stabilizes a catalytic intermediate, is also visible on the left. (*e*) Occlusion of the active-site residues by Asn114 and Asn261. We propose that their proximity to each other is a result of interactions with the propeptide and assists in the prevention of binding of the substrate. Upon removal of the propeptide, the distance may be lengthened slightly, opening the active site.

The high-resolution structure was solved in the monoclinic space group *P*2_1_ to 1.4 Å resolution with two molecules in the crystallographic asymmetric unit. It was refined to final *R*_cryst_ and *R*_free_ values of 13.8 and 16.9%, respectively, and also contained two calcium ions, two sulfate ions, eight PEG molecules, six glycerol molecules and 927 water molecules, with an estimated solvent content of 43.8%. Calcium ion identities were determined by their ability to fill electron density and were confirmed through coordinate bond lengths (Harding, 2004 ▶; Zheng *et al.*, 2008 ▶). Overall, 96.1% of the residues are in the preferred regions of the Ramachandran plot, with 3.9% in the allowed regions and no outliers. The crystallographic statistics are summarized in Table 1 ▶. Poor electron density was observed between residues Gly58 and Tyr63, although we were able to interpret this part of the structure with a fair degree of certainty; little to no density was observed between Lys81 and Tyr89, so this region was not built in the structure (Fig. 3 ▶ *a*).

### 3.2. Propeptide   

The propeptide largely consists of loop regions with a central helix (α1) and short β-strand (β1). The poorly defined region was determined to contain a short helix in chain *B* but not in chain *A*: our secondary-structure numbering assumes that this helix is not present.

**Figure 4:**
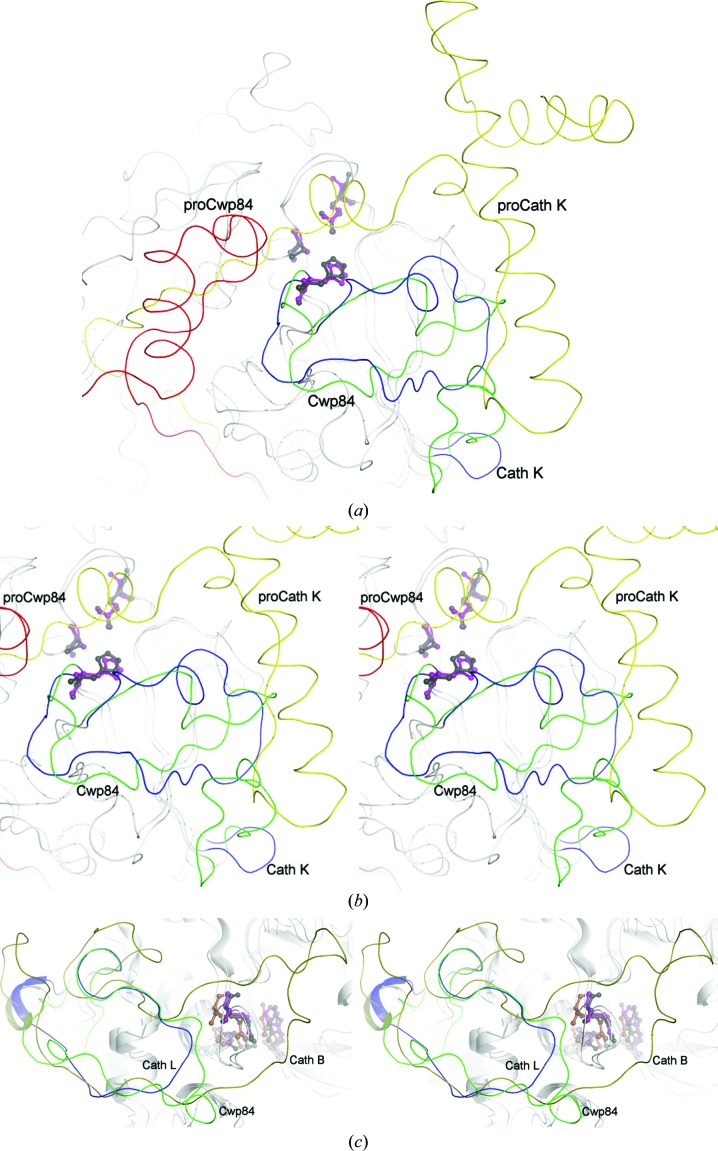
Structural comparisons between Cwp84 and other cysteine proteases. (*a*) Comparison of cysteine protease propeptides and prosegment binding loops (PBLs). Structures are rendered as coils for simplicity. Overview of the whole region, showing the Cwp84 propeptide in red and cysteine protease domain in green, and the cathepsin K (PDB entry 7pck; cathepsin L-like; Sivaraman *et al.*, 1999 ▶) propeptide in yellow with the cysteine protease domain in blue; Cwp84 active-site residues are shown in purple. Active-site residues of Cwp84 are shown in magenta and those of cathepsin K are shown in black. Both propeptides cover the active-site groove, shown on the left. Cathepsin propeptides wrap around the protein, interacting with the PBL and forming a conserved helix, while Cwp84 folds back on itself and wraps around the lectin-like domain, leaving the top of the active site considerably more exposed. (*b*) Cross-eyed three-dimensional view of the PBL. The usually conserved α-­helix and short β-sheet are not present in Cwp84, with the whole chain rotated roughly 90°. A turn or short loop below the PBL is replaced by a 16-residue loop that occupies the space normally taken up by the propeptide. (*c*) Cross-eyed three-dimensional comparison of cysteine protease occluding loop regions. Cwp84 is shown in green, cathepsin L (PDB entry 1cjl; Coulombe *et al.*, 1996 ▶) in blue and cathepsin B (PDB entry1pbh; Turk *et al.*, 1996 ▶) in olive. The active-site residues of Cwp84 (Gln110, C116A and His262) are shown in purple, those of cathepsin L are shown in black and those of cathepsin B in brown. The fold of cathepsin L is well conserved; many cathepsin L-like proteases will superpose very closely in this region. The relatively short loop does not affect interactions with the active site. Cathepsin B-like proteases have a significantly longer, more variable loop that controls substrate specificity and confers carboxypeptidase activity. The equivalent loop in Cwp84 is closer to that of cathepsin L-like proteases but is slightly longer and could be involved in substrate binding.

The N-terminal portion of the Cwp84 propeptide (His33–Gly65) wraps around the lectin-like domain (Figs. 1 ▶ *b* and 1 ▶ *c*) and does not exhibit similarities to propeptides from other papain proteases, which commonly form a small globular domain covering the top of the active site and are stabilized by a β-sheet formed by interaction with the prosegment binding loop (PBL; Figs. 4 ▶ *a* and 4 ▶ *b*). This novel conformation leaves the S′ end of the active-site groove (the portion of the active-site groove that interacts with the peptide substrate after the scissile bond, based on the active-site nomenclature of proteases; Sajid & McKerrow, 2002 ▶; Schechter & Berger, 1967 ▶) significantly more accessible than in other cysteine proteases. Nevertheless, the catalytic residues are partially occluded by Asn114 and Asn261 (Fig. 3 ▶ *e*).

The C-terminal portion of the propeptide (Val66–Arg79) forms an extended loop that sits in the active-site cleft. The poorly defined helix (found only in chain *B*) that precedes this loop is considerably removed from the active site, around 7–8 Å away from its location in both cathepsin L and cathepsin B (Fig. 4 ▶ *c*). Residues Asn64–Ile67 form a hydrogen-bond network with the cysteine protease domain. These interactions are mainly with Met160–Ser164, but hydrogen bonds are also formed to Asn114 and Leu260. After this, the propeptide enters the active-site groove, with Pro70–Glu72 forming hydrogen bonds to the N-terminal part of the propeptide. Thr76–Arg79 form a large number of hydrogen bonds to the lectin-like domain and the cysteine protease domain. Close interactions between the propeptide and the cysteine protease domain are seen in many other proteins (Coulombe *et al.*, 1996 ▶; Sivaraman *et al.*, 1999 ▶), but as the lectin-like domain is a newly observed feature of a cysteine protease, so too are its interactions with the propeptide.

There are usually two main points on a cysteine protease to which its propeptide is anchored: the surface-exposed PBL (prosegment-binding loop), which the propeptide of Cwp84 does not approach, and the S_2_ subsite of the active-site cleft, which is occupied by a residue that mimics the substrate (Coulombe *et al.*, 1996 ▶; Sivaraman *et al.*, 1999 ▶). Interestingly, in Cwp84 this latter position is occupied by Val66 from the propeptide, while the P_2_ residue of SlpA is usually lysine. Although Val66 is able to interact with the S_2_ subsite through van der Waals interactions, the shorter, hydrophobic side chain does not enter the negatively charged pocket (Fig. 3 ▶ *d*). Given the apparent lack of PBL stabilization and the shorter Val66, the propeptide is likely to be stabilized through other multi-domain interactions.

Treatment of the purified recombinant GST-Cwp84_33–497_ protein (78.5 kDa) with trypsin was found to result in the loss of approximately 33.5 kDa, giving a single band of 45 kDa. The mass of this protein, as confirmed by mass-spectrometric analysis, was 45 058 Da, and therefore the loss of 33.5 kDa from the protein is consistent with removal of the pro­region and GST. The N-terminal sequencing determined that the remaining 45 kDa protein had an N-terminus of SSVAY, confirming that the proregion up to Ser92 had been removed. These data suggest that the proregion is folded in Cwp84_33–497_ in such a way that it is accessible for cleavage by trypsin and that artificial maturation has replicated the removal of the proregion up to Ser92 as observed in *C. difficile* (ChapetónMontes *et al.*, 2011 ▶; de la Riva *et al.*, 2011 ▶).

### 3.3. Cysteine protease domain   

The overall fold of the cysteine protease domain of Cwp84 is similar to those of other papain proteases, particularly cathepsin L-like proteases. A *DALI* structural similarity search (Holm & Rosenström, 2010 ▶) indicates that it shares the highest level of similarity with *Toxoplasma gondii* cathepsin L (*Z* = 23.9, sequence identity 20%; PDB entry 3f75; Larson *et al.*, 2009 ▶), rhodesain from *Trypanosoma brucei* (Z = 23.6, sequence identity 21%; PDB entry 2p7u; Kerr *et al.*, 2009 ▶) and cruzipain from *T. cruzi* (*Z* = 23.5, sequence identity 19%; PDB entry 4klb; Wiggers *et al.*, 2013 ▶).

The cysteine protease domain exhibits a typical, approximately U-shaped fold with two subdomains flanking the central active-site cleft, one formed by a twisted antiparallel β-sheet containing four β-strands (β4, β6, β7 and β8), one helix (α5) and several loop regions, and the other formed by a central 15-residue-long α-helix (α2) surrounded by two short α-helices (α3 and α4), an antiparallel β-sheet containing two strands (β3 and β9) and several loop regions (Fig. 2 ▶).

The active site of the cysteine protease domain of Cwp84 is similar to those of other cysteine proteases with regard to the positions of the active-site residues Cys116 (mutated to alanine in the present study), His262 and Gln110. Asn287, which has previously been suggested to be an active-site residue (Savariau-Lacomme *et al.*, 2003 ▶), is not located within the active site.

### 3.4. Lectin-like domain   

We have discovered that the approximately 170-residue ‘linker’ region between the cysteine protease domain and the first cell-wall-binding domain in full-length Cwp84 forms a single domain (residues 335–497) consisting of 13 β-strands (β10–β22), eight of which form a twisted antiparallel β-sandwich with a hydrophobic core. Proteins with similar folds to this domain were determined using a *DALI* search. The majority of the most similar results were carbohydrate-binding proteins, including *Clostridium perfringens* α-*N*-acetylgluco­saminidase (*Z* = 8.1, sequence identity 14%; PDB entry 2vcc; Ficko-Blean *et al.*, 2008 ▶), a sialidase from *Micromonospora viridifaciens* (*Z* = 8.0, sequence identity 11%; PDB entry 2bzd; Newstead *et al.*, 2005 ▶) and a noncatalytic carbohydrate-binding module from *Clostridium thermocellum* (*Z* = 7.7, sequence identity 8%; PDB entry 2yb7; Montanier *et al.*, 2011 ▶); we therefore designate this domain the ‘lectin-like’ domain. There were, however, a significant number of non­carbohydrate-binding results, including E3 ubiquitin ligases such as *Mus musculus* MYCBP2 (*Z* = 9.5, sequence identity = 13%; PDB entry 3hwj; Sampathkumar *et al.*, 2010 ▶), human DNA-repair protein XRCC1 (*Z* = 8.2, sequence identity 10%; PDB entry 3k77; Cuneo & London, 2010 ▶) and *Chlamydomonas reinhardtii* intraflagellar transport protein 25 (*Z* = 8.1, sequence identity 9%; PDB entry 2yc4; Bhogaraju *et al.*, 2011 ▶). The lectin-like domain contains a calcium ion coordinated by Leu339, Glu448, Lys460, Asn487 and two water molecules. Most of the conserved residues within the lectin-like domain are found within β-strands, are hydrophobic or bind calcium (Fig. 2 ▶). This indicates that the structure and potentially the function of the lectin-like domain is conserved amongst these proteins, of which we believe this to be the first report.

The lectin-like domain contains a hydrophobic core that opens at the surface of the protein, producing a hydrophobic pocket formed by residues Ile347, Ile468, Ile477 and Phe483. Interestingly, both Leu36 and Val39 from the propeptide insert into this pocket, with Lys34 hydrogen bonding to Thr479, suggesting that these interactions may provide stabilizing roles through hydrophobic interactions.

**Figure 5:**
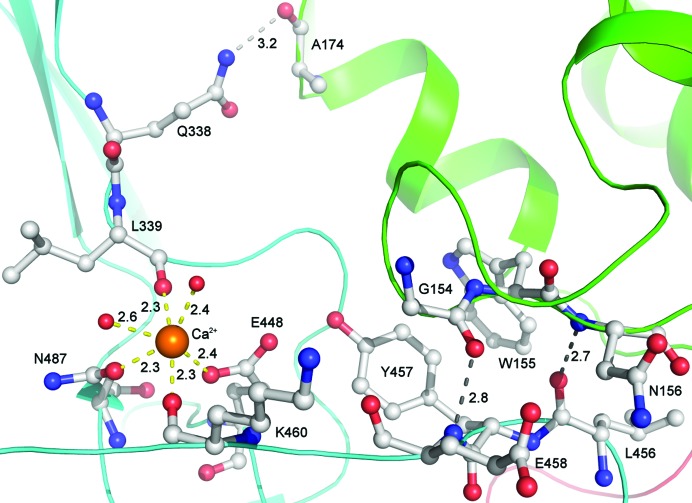
Calcium ion coordination by the lectin-like domain and two water molecules. Nearby hydrogen bonds between the lectin-like domain and the cysteine protease domain (two of three sets of charge-based interactions between the two domains) are also shown. Domains are coloured according to Fig. 1 ▶, coordinate bonds are shown in yellow and hydrogen bonds are shown in grey. Calcium ion coordination brings together distant parts of the primary structure and is likely to be essential for correct folding.

The cysteine protease domain and the lectin-like domain also have interaction points between the two domains at three locations: Gln338, Leu457–Glu458 (Fig. 5 ▶) and Tyr408–Asn413. The glutamine residue at position 338, which is highly conserved in the *BLASTP* results (Fig. 2 ▶), forms an isolated hydrogen bond; Leu457–Glu458 form main-chain hydrogen bonds, while Tyr408–Asn413 make both main-chain and side-chain interactions.

Two of the three regions where the lectin-like and cysteine protease domains interact (Gln338 and Leu457–Glu458) are both sequentially and spatially close to the calcium ion-binding site (formed by Leu339, Glu448, Lys460 and Asn487).

## 4. Discussion   

In this study, we have elucidated the structure of residues 33–497 of Cwp84, the surface-associated cysteine protease of *C. difficile* which plays a key role in the maturation of the S-layer protein SlpA. The high-resolution structural data presented here will improve the understanding of the role of Cwp84 in S-layer biogenesis. In addition, the discovery of a newly identified calcium-binding lectin-like (putative carbohydrate-binding) domain raises exciting possibilities with regard to the potential role(s) that this region may have in S-layer biogenesis in *C. difficile* and also in other species, such as those presented in Fig. 2 ▶. We also compared the structure of the cysteine protease domain (C1A family) of Cwp84 with those reported for the cysteine protease domains (C80 family) from the large clostridial toxins of *C. difficile* (TcdA and TcdB; Pruitt *et al.*, 2009 ▶; Shen *et al.*, 2011 ▶) and found no detectable structural similarity between the two classes of cysteine protease structures.

We observed that the cysteine protease domain retains a strong structural similarity to other papain-family enzymes, namely the cathepsins, particularly cathepsin L. However, significant differences exist between Cwp84 and structurally similar proteases.

Cathepsin B-like proteases possess a long loop, known as the occluding loop, which partially blocks the S end of the active site. This allows greater endopeptidase substrate specificity and also confers carboxypeptidase activity on the protein, with a conserved HH motif in the occluding loop binding the substrate at the S_2_′ position (Sajid & McKerrow, 2002 ▶). In the same position, cathepsin L-like proteases possess a much shorter loop that does not block the active site, allowing the cleavage of a broader range of substrates (Coulombe *et al.*, 1996 ▶). The equivalent loop in Cwp84 (found between α4 and β3) is closer to that of cathepsin L-like proteases. Although slightly longer than the usually well conserved fold, it is much shorter than the occluding loop found in cathepsin B-like proteases and does not contain the HH motif (Fig. 4 ▶ *c*). This loop is poorly conserved among closely related proteins (Fig. 2 ▶) and thus may be involved in substrate selectivity.

The loop formed between helix 3 and helix 4, which forms one side of the active-site cleft and has a position that is well conserved in other cysteine proteases, is roughly 3–4 Å further away from the active site than usual. This presents a deeper active-site cleft, which may be important for substrate binding and specificity. This loop also contains two residues that form a β-bridge with the lectin-like domain, forming one of the three contact points between the two domains (Fig. 5 ▶). The active-site cleft then continues in the S direction with one side formed by the cysteine protease domain and the other by the lectin-like domain, which, as it has not been observed in other cysteine protease structures, gives the S end of the active site a significantly different shape (Fig. 3 ▶).

Moreover, in papain proteases, a residue above the S_2_ position of the active site has been shown to play a significant role in determination of substrate specificity: this position is occupied by Ser205 in papain, Ala214 in cathepsin L and Glu245 in cathepsin B (Sajid & McKerrow, 2002 ▶). In Cwp84, S_2_ selectivity is likely to be controlled by Asp320, which, along with Ser235, Thr317 and Asp318, forms a negatively charged pocket which is likely to stabilize the binding of the P_2_ lysine residue usually found in SlpA (Fig. 3 ▶ *d*). Indeed, mutation of the P_2_ lysine to alanine has been shown to abolish the cleavage of an SlpA fragment by Cwp84 in co-expression studies, suggesting its significance in SlpA cleavage (Dang *et al.*, 2010 ▶).

We believe the lectin-like domain to be a newly observed feature of cysteine proteases, particularly those from Clostridiales. It bears some resemblance to the jelly-roll domain of the clostridial serine protease CspB, in that both are β-sandwiches that are closely associated with a protease domain (Adams *et al.*, 2013 ▶). The two could possess similar functions, namely conferring resistance to degradation, positioning the prodomain for cleavage and assuring the correct conformation of the protease domain. Even though the cores of the lectin-like domains appear to have a similar structure, there are significant changes (resulting in a large root-mean-square deviation) in the positioning of the β-strands, including the loop regions. Further experimental studies will be required to confirm the role(s) of the lectin-like domain in Cwp84.

Interestingly, lectin-like interactions have been suggested to be involved in S-layer array formation, particularly with regard to the linkage between the S-layer subunits and secondary cell-wall polymers (SCWPs; Ferner-Ortner *et al.*, 2007 ▶; Sára *et al.*, 1998 ▶; Sára & Sleytr, 2000 ▶).

The carbohydrate-binding region seen in many of the *DALI* results does not appear to be present in Cwp84, indicating that if the lectin-like domain does bind carbohydrates, it does so using a different interface. IFT25 (intraflagellar transport protein 25) has a fold almost identical to that of sialidases, but the carbohydrate-binding region is replaced by a region that interacts with a helix from IFT27 to form the IFT25/27 complex (Bhogaraju *et al.*, 2011 ▶). In Cwp84, the equivalent region interacts with the propeptide. If the Cwp84 lectin-like domain does bind carbohydrates (or a different cofactor) in this region, it is possible that the propeptide prevents binding. It is also not unreasonable to assume that despite its similarity to carbohydrate-binding proteins, the lectin-like domain of Cwp84 may assume a completely different function.

We believe the close interactions between the cysteine protease domain, the lectin-like domain and the propeptide are likely to be essential to the initial folding of the protein and will mediate substrate binding and specificity.

## 5. Conclusions   

We have determined the structure of the Cwp84 cysteine protease domain with its bound propeptide and a newly discovered lectin-like domain. The propeptide sits in the active-site groove and wraps itself around the lectin-like domain, closely interacting with both domains, a feature that is likely to be important in the initial folding of the protein. The cysteine protease domain, although similar to many previously determined cathepsin L-like structures, bears significant differences; namely, the active-site groove is deepened by the lectin-like domain, the PBL is not present and the would-be occluding loop is slightly longer. The lectin-like domain bears a similar β-sandwich fold to that seen in many carbohydrate-binding proteins, but it is currently unclear what function it possesses. If it does bind a carbohydrate, it is possible that the lectin-like domain may be involved in substrate recognition or attachment to the cell wall, resulting in correct orientation of the cysteine protease domain for cleavage of SlpA.

Further structural and functional studies are necessary to elucidate the exact mechanism of Cwp84-mediated SlpA cleavage and how this contributes to overall S-layer biosynthesis. Given the likely key role of the *C. difficile* surface in growth and colonization, the potential development of anti-colonization inhibitors or vaccines is significantly aided by structural data such as that presented here.

## Supplementary Material

PDB reference: Cwp84, 4ci7

## Figures and Tables

**Table 1:** X-ray crystallographic statistics Values in parentheses are for the outer shell.

	Native	Peak	Inflection	High remote	Low remote
Energy (eV)	12658	12660	12656	12770	12550
Wavelength ()	0.9795	0.9793	0.9796	0.9717	0.9879
Space group	*P*2_1_	*P*2_1_	*P*2_1_	*P*2_1_	*P*2_1_
Unit-cell parameters					
*a* ()	50.9	50.9	51.0	50.7	51.3
*b* ()	73.5	73.1	73.2	73.0	73.6
*c* ()	125.6	125.4	125.7	125.7	125.4
= ()	90.0	90.0	90.0	90.0	90.0
()	93.6	93.5	93.5	93.9	93.1
Resolution range ()	48.21.40	29.72.10	29.72.10	29.62.50	29.31.94
*R*_merge_ (%)	9.9 (25.6)	32.2 (57.5)	32.5 (57.6)	18.5 (54.2)	13.9 (81.5)
*I*/(*I*)	16.0 (4.2)	16.9 (7.7)	16.0 (6.3)	14.5 (5.1)	9.2 (2.0)
Completeness (%)	93.9 (65.3)	100.0 (100.0)	100.0 (100.0)	99.9 (100.0)	99.4 (96.4)
Total No. of reflections	810986	1120802	945054	333693	489672
Unique reflections	170213	52790	54120	31917	68579
Multiplicity	4.8 (2.4)	20.8 (20.5)	17.5 (14.0)	10.5 (10.2)	7.1 (5.6)
Anomalous completeness (%)	76.5 (25.8)	100.0 (100.0)	100.0 (100.0)	99.9 (100.0)	98.2 (90.7)
Anomalous multiplicity	2.2 (0.7)	10.5 (10.2)	8.8 (6.9)	5.3 (5.1)	3.6 (2.9)
CC_anom_ 0.3 ()	N/A	3.8	4.4	5.5	N/A
Wilson *B* factor (^2^)	9.8	13.4	17.4	24.7	21.7
*R*_cryst_/*R*_free_ (%)	13.8/16.9				
Average *B* factor (^2^)					
Overall	18.4				
Protein	16.7				
Ligand	36.6				
Solvent	29.8				
R.m.s. deviations					
Bond lengths ()	0.008				
Bond angles ()	1.340				
Ramachandran plot statistics					
Preferred (%)	96.1				
Allowed (%)	3.9				
Disallowed (%)	0				
PDB code	4ci7				
